# *Salvia ceratophylla* L. from South of Jordan: new insights on chemical composition and biological activities

**DOI:** 10.1007/s13659-020-00259-9

**Published:** 2020-08-27

**Authors:** Mohammad Sanad Abu-Darwish, Célia Cabral, Zulfigar Ali, Mei Wang, Shabana I. Khan, Melissa R. Jacob, Surendra K. Jain, Babu L. Tekwani, Fazila Zulfiqar, Ikhlas A. Khan, Hatem Taifour, Lígia Salgueiro, Thomas Efferth

**Affiliations:** 1 Department of Basic and Applied Sciences, Al-Balqa Applied University, Al-Salt, 19117 Jordan; 2 National Center for Natural Products Research, School of Pharmacy, University of Mississippi, University, MS 38677 USA; 3 Coimbra Institute for Clinical and Biomedical Research (iCBR), Faculty of Medicine, University of Coimbra, 3000-548 Coimbra, Portugal; 4 Center for Innovative Biomedicine and Biotechnology (CIBB), University of Coimbra, 3000-548 Coimbra, Portugal; 5 Centre for Functional Ecology, Department of Life Sciences, University of Coimbra, Calçada Martim de Freitas, 3000-456 Coimbra, Portugal; 6 School of Geosciences, University of Edinburgh, 219 Crew Building, King’s Buildings, Edinburgh, EH9 3FF UK; 7 CIEPQPF/Faculty of Pharmacy, University of Coimbra, Health Sciences Campus, Azinhaga de S. Comba, 3000-548 Coimbra, Portugal; 8 Department of Pharmaceutical Biology, Institute of Pharmaceutical and Biocmedical Sciences, Johannes Gutenberg University, Staudinger Weg 5, 55128 Mainz, Germany

**Keywords:** Lamiaceae, Essential oil, Chemical composition, Cytotoxicity, Anti-inflammatory activity, Neglected diseases

## Abstract

In Jordan, *Salvia ceratophylla* L. is traditionally used in the treatment of cancer, microbial infections, and urinary disorders. This study aimed: (1) to chemically characterize *S. ceratophylla* essential oil (EO) from South Jordan, by gas chromatography (GC) and gas chromatography-mass spectrometry (GC–MS); and (2) to evaluate in vitro the cytotoxic, anti-inflammatory, and antiprotozoal activities of the EO, it’s predominant components, and the hexane (A), ethyl acetate (B), methanol (C) and crude-methanol extracts (D). The analysis revealed that the EO has 71 compounds, with linalool (54.8%) as main constituent. Only the hexane extract (A) showed some cytotoxic activity against SK-MEL, KB, BT-549, SK-OV-3, LLC-PK1 and VERO cells lines with IC_50_ between 60 and > 100 µg/mL. The EO inhibited NO production (IC_50_ 90 µg/mL) and NF-κB activity (IC_50_ 38 µg/mL). The extracts A, B, and D inhibited NO production and NF- κB activity with IC_50_ between 32 and 150 µg/mL. Linalool considerably inhibited NO production (IC_50_ 18 µg/mL). The extracts tested did not exhibit antileishmanial activity. Regarding antitrypanosomal activity, the EO exhibited significant results with IC_50_ 2.65 µg/mL. In conclusion, Jordan *S. ceratophylla* EO represents a rich source of linalool and bears a promising therapeutic potential for further antitrypanosomal drug development.

## Introduction

*Salvia ceratophylla* L. (Arabic: *Lusan Al-haea*, English: horn-leaved sage) is a biennial lemon-scented herb and one of the 19 indigenous *Salvia* species native in Jordan [1]. This species grows in diverse Jordanian geographic areas, such as Mafraq, Ajlun, Ma'an, Tafila and Dana [1] and Wadi Musa [2]. It is also found in several Asian-temperate regions, including Caucasus, Transcaucasus, Middle Asia (Turkmenistan), and Near East **(**Afghanistan, Iran, Iraq, Lebanon, Syria, Palestine, Israel, and Turkey) [3, 4].

*S. ceratophylla* is used in traditional medicine of the Middle East against various ailments, e.g*.* inflammation, fungal, and nociceptive diseases [4–6]. In Middle Eastern countries such as Jordan, Lebanon, and Syria, the herbalists prescribe *Salvia* species as boiled tea to relieve abdominal pain, headaches, stomachaches, and to treat cancer, microbial infections, asthma, coughs and other pulmonary and urinary disorders [7]. It is also recommended in folk medicine to disinfect homes after sickness [8]. Several *Salvia* species and their essential oils are used in various traditional medicines to treat malaria [9, 10] and leishmaniasis [11].

Despite the composition of *S. ceratophylla* essential oil (EO) grown in various habitats of the Near East countries has been analyzed and a wide variation in their composition has been reported [3, 12–14], there is only one report on the composition of *S. ceratophylla* EO originated from the middle of Jordan [15].

Few reports showed the biological activities of *S. ceratophylla* extracts from various locations in the Middle East. Some *S. ceratophylla* extracts revealed cytotoxic activity against some types of cancer cell lines [16, 17].

The antioxidant activity of *S. ceratophylla* originated from Turkey and Iran has been assessed by different methods. Gürsoy et al. [3] showed the antioxidant activity of essential oil of *S. palaestina* and *S. ceratophylla* from Turkey. The *S. ceratophylla* chloroform extract inhibited the activity of butyrylcholine esterase [18]. Among 9 *Salvia* species from Iran, the methanolic extract of *S. glutinosa* and *S. ceratophylla* revealed the strongest antioxidant activity [17]. The anti-inflammatory activity of the EO from *Salvia* species is mediated by the inhibition of nitric oxide (NO) production and nuclear kappa B (NF-κB) activation [10]. Linalool and linalyl acetate, the major volatile constituents of the EO of several aromatic plants exhibit in vivo strong anti-inflammatory activity against Carrageenin-induced edema [19].

The extracts and EO of some other *Salvia* species showed antiprotozoal activity against malaria, leishmaniasis and human African trypanosomiasis [20–22]. The hydroalcoholic extract of *S. officinalis* from Algeria completely inhibited the growth of *Leishmania major* [23]. Furthermore, linalool exhibited antileishmanial activity in a concentration-dependent fashion [24, 25]. Caryophyllene showed anti-protozoal activity against *Leishmania donovani* [26] and *Trypanosoma brucei* [27].

Only a few studies reported the biological activities of *S. ceratophylla* from Jordan. However, the anti-inflammatory activity of hexane, ethyl acetate and methanol extracts obtained from the aerial parts of Jordanian *Salvia* species have been evaluated by inhibition of croton oil-induced mouse ear edema [6]. Kasabri et al. [5] studied the inhibitory activity of the aqueous extracts of *S. ceratophylla* and other *Salvia* species grown in Jordan against pancreatic triacylglycerol lipase, α-amylase and α-glucosidase. *S. ceratophylla* exhibited cytotoxic efficacy against CaCo2 cells with an IC_50_ value of 9.2 ± 0.5 μg/mL.

However, there is limited information about the chemical composition and biological activities of medicinal plants from Jordan flora. No studies have been carried out yet on *S. ceratophylla* EO from Dana Biosphere Reserve in southern Jordan and to the best of our knowledge, there are no previous reports on the antiprotozoal activity of this plant. Thus, the aim of this work was to evaluate for the first time the chemical composition of *S. ceratophylla* EO grown in Dana Biosphere Reserve (Southern Jordan), as well as, to assess the cytotoxic, antileishmanial, and antimalarial activities of its essential oil and other extracts.

## Materials and Methods

### Plant Material

Aerial parts of *S. ceratophylla* were collect during the spring season, throughout Dana Biosphere Reserve (South of Jordan). The voucher specimens were identified by Mr. Hatem Taifour (Researcher at School of GeoSciences, University of Edinburgh Edinburgh EH9 3FF, U.K.) and were deposited in the Herbarium of Shoubak University College, Al-Balqa Applied University, Jordan under the number #0009- Sc-DSh-2015 and in the Herbarium of National Center for Natural Products Research, School of Pharmacy, University of Mississippi, USA under the number #18,330. The plant material for extraction was air dried and crushed into small pieces with a hammer mill before extraction.

### Isolation of Extracts

#### Isolation of Essential Oil

To isolate EO, the crushed aerial parts of *S. ceratophylla* were hydro distillated during 3 h with a Clevenger-type apparatus, according to the European Pharmacopoeia [28]. The oil obtained was dried over anhydrous sodium sulphate and kept at 4 ºC until analyzed and tested.

#### Extraction of Non-polar and Polar Extracts

The hexane, ethyl acetate, and methanol extracts from the crude areal parts of *S. ceratophylla* were obtain as follows: powdered plant material (600 g) was soaked with hexane followed by ethyl acetate, and then methanol for 48 h for each solvent in ratio of 500 mL per 100 g. The process of extraction for each solvent was repeat several times to ensure complete extraction of the compounds. The obtained extracts for each solvent were combine together and subject to rotary evaporation at 40 °C. The evaporation led to dry hexane (A), ethyl acetate (B), and methanol (C) extracts with yields of 4.21%, 1.77% and 6.93%, respectively.

#### Extraction of Crude Methanol Extract

A sample of 50 g of powder plant materials was soaked in 250 mL of methanol for 48 h. The process of extraction was repeat several times to ensure complete extraction of the compounds. The obtained methanol extracts were filter combined and subject to rotary evaporation at 40 °C. The obtained crude methanol dry extract (D) yielded 18.96%. All extracts (A-D) were freeze-dry and stored at 4 °C for further analyses.

### Chemical Composition

#### Chemicals

GC-grade n-hexane (> 99%) and the reference standards were purchased from Sigma-Aldrich or isolated in the National Center for Natural Products Research, University of Mississippi. A mixture of n-alkanes (C_8_H_18_–C_23_H_48_) was use in the determination of retention indices and were purchased from PolyScience Corp.

#### Gas Chromatography-Mass Spectrometry (GC–MS)

Gas chromatography analysis was performed in an Agilent 7890B GC equipped with a 5975C quadruple mass spectrometer and a 7693-auto sampler. Separation was achieve using an Agilent J&W HP-1 (100% dimethyl-polysiloxane) fused silica capillary column (60 m × 0.25 mm I.D. × 0.25 µM film thickness). Helium was the carrier gas at a constant flow rate of 1.5 mL/min. Oven temperature program: 50 °C hold for 2 min, after 50–170 °C at 2 °C/min, 170–250 °C at 6 °C/min (hold 10 min). The injector temperature was 250 °C, the injection volume was 0.5 µL and a split ratio of 50:1. The EO was diluted in n-hexane (0.5% v/v) prior to GC/MS analysis.

Mass spectra were record at 70 eV at a scan mode from m/z 40 to 500. The transfer line and the ion source temperatures were 260 °C and 230 °C, respectively. Data acquisition was made with Agilent MSD Chemstation (F.01.03.2357).

#### Identification of Individual Components

Compound identification was carried out through comparison of the spectra with the database (Wiley and NIST) using a probability-based matching algorithm. Further identification was based on the relative retention indices compared with reference standards and literature data [29].

### Cytotoxic activity

All cell lines used in this study were purchase from American Type Culture Collection (ATCC, Rockville, MD). The cytotoxic activity of the EO, extracts isolated from *S. ceratophylla* and pure compounds was determined in vitro against various human cancer cells: skin malignant melanoma (SK-MEL), human KB carcinoma (KB), breast ductal carcinoma (BT-549), ovarian carcinoma (SK-OV-3), and in two non-cancerous mammalian kidney cell lines (LLC-PK1 and VERO).

Cells were seed at a density of 25,000 cells/well and incubated during 24 h. The tested EO, extracts and pure compounds were added separately at studied concentrations and cells were re-incubated during 48 h. After incubation time, the viability of cells was assessed by using Neutral Red Dye as described [30]. DMSO was use as negative control and doxorubicin as positive control.

### Anti-inflammatory Activity

#### Inhibition of Cellular Oxidative Stress Assay

The cellular antioxidant activity of *S. ceratophylla* EO and other extracts was measured in human HepG2 hepatoma cells This assay determines the ability of test samples to prevent intracellular generation of peroxyl radicals in response to 2,2- azobis (2-amidinopropane) dihydrochloride (ABAP, Sigma-Aldrich, St Louis, MO, USA).

HepG2 cells were culture in Dulbecco's Modified Eagle Medium (DMEM) supplemented with 10% fetal bovine serum (FBS), 50 units/mL penicillin and 50 µg/mL streptomycin. Cells were seed in 96-well plates (60,000 cells/well) and incubated for 24 h. The tested samples were diluted in serum free medium containing 25 µM 2^′^,7′, dichoroflouresceine diacetate (DCFH-DA, Invitrogen, Carlsbad, CA, USA). The cells were wash with PBS and treated with test samples during 1 h. The medium containing tested samples was removed, ABAP (600 µM) was added and the plate was immediately placed on a Spectra Max plate reader for kinetic reading every 5 min during 1 h (37 ºC, emission at 538 nm and excitation at 485 nm). Quercetin (Sigma-Aldrich) was use as positive control. To calculate the percentage of decrease in oxidative stress, the Area Under the Curve (AUC) of fluorescence versus time was used.

$${\text{Decrease in oxidative stress }}\left( \% \right) \, ={{\text{AUC control}}}^{{{1}00 - \, [{\text{AUC tested sample}} \times { 1}00]}} \,$$

#### Nitric Oxide (NO) Measurement

The NO inhibition by EO, extracts of *S. ceratophylla* and pure compounds, was assess by the following methodology [31]. Briefly, mouse macrophage cells (RAW 264.7) were culture in phenol red free RPMI medium with 10% bovine calf serum and 100 U/mL penicillin and 100 µg/mL streptomycin.

Cells were seed in 96-well plates (50,000 cells/well) and incubated during 24 h. The tested samples were dilute in serum free medium, incubated during 30 min, then lipopolysaccharide (LPS, 5 µg/mL) was added and cells were incubated for further 24 h. The level of nitrite in the cell supernatant was measure by using Griess reagent. The inhibition of nitrite production by the tested samples was calculate in comparison to the vehicle control. Parthenolide (Sigma-Aldrich) was use as positive control. IC_50_ values were obtain from dose curves.

#### Assay for Inhibition of NF-κB Activity

The inhibition activity of tested samples against NF-κB was evaluate as described [31]. The SW 1353 human chondrosarcoma cell line was culture in a mixture of Dulbecco's Modified Eagle's Medium (DMEM) and Ham's F-12 Nutrient **(**DMEM/F12**)** supplemented with 10% FBS, 100 U/Ml penicillin and 100 µg/mL streptomycin. Cultured cells were washed in antibiotic and FBS-free DMEM/F12 medium and then re-suspended in FBS-free DMEM/F12 medium containing 2.5% FBS. NF-κB luciferase plasmid construct was added to the cell suspension in a concentration of 50 µg/mL and incubated for 5 min at 25ºC. The cells were transfected by electroporation at 160 V and one 70 ms pulse using BTX disposable cuvettes (model 640 in a BTX Electro Square Porator T 820—BTX I, San Diego, CA, USA), Transfected cells were plate in 96-well plates at a density of 1.25 × 10^5^cells per well in 200 µL of DMEM/F12 containing 10% FBS and antibiotics. After incubation for 24 h, the cells were treated with test samples for 30 min and then induced with PMA (70 ng/mL) for 8 h. The inhibition of NF-κB activity by EO, other extracts, and pure compounds was calculated in comparison to vehicle control. The IC_50_ values were obtain from the dose curves. Parthenolide was use as positive control.

### Antileishmanial and Antitrypanosomal Activities

#### Sample Preparation

EO and extracts (A-D) at a stock concentration of 20 mg/mL were dilute to 4 mg/mL with DMSO. The obtained solutions (4 mg/mL) were four-fold diluted with incomplete RPMI medium to 1 mg/mL. Pure compounds at a stock concentration of 2 mg/mL in DMSO were diluted four-fold with incomplete RPMI medium to 0.5 mg/mL.

#### In Vitro Antileishmanial and Antitrypanosomal Assays

The extracts and pure compounds (highest concentrations 20 µg/mL for extracts and 10 µg/mL for pure compounds) were subject to different tests, namely *Leishmania donovani* promastigote assay, *L. donovani* axenic amastigote assay*, L. donovani* THP1 macrophage amastigote assay*, Trypanosoma brucei* trypamastigotes assay and THP1 toxicity assay. Alamar Blue assay was perform as previously described by other authors [32, 33].

These assays have been adapted to 384-well micro-plate format. A 3–4 days old culture of *Leishmania donovani* promastigotes harvested in the exponential growth phase was diluted with RPMI medium to 1 × 10^6^ cells/mL for promastigote assays. A 3–4 days old culture of *Leishmania donovani* axenic amastigotes was diluted with RPMI medium to 2 × 10^6^ cells/mL for axenic amastigote assays. A 2 days old culture of *Trypanosoma brucei* harvested in the exponential phase was diluted with Iscove's Modified Dulbecco's Medium (IMDM) to 5 × 10^3^ cells/mL for the antitrypanosomal assay.

Samples were added to the *L. donovani* promastigotes, *L. donovani* axenic amastigotes or *T. brucei* trypamastigotes cultures. The extracts were initially test at single concentrations of 20 mg/mL for primary screening. The active extracts and pure compounds were test at three or six concentrations ranging from 10 to 0.0032 µg/mL. The plates were incubated at 26 °C for 72 h (37 °C for axenic amastigotes and *T. brucei* trypamastigotes) and growth of cultured parasites was determined by the Alamar Blue assay [32 33].

The extracts and single compounds were also test against *L. donovani* intracellular amastigotes in THP1 cells employing a recently developed parasite-rescue and transformation assay [15].

### THP1 Toxicity Assay and Macrophage Amastigote Assay

A 4 days old THP1 cell culture harvested in the exponential growth phase was diluted with RPMI medium to 2.5 × 10^5^ cells/mL. PMA was added to a final concentration of 25 ng/mL. A PMA treated culture was dispense in experimental culture plates and incubated overnight at 37 °C in a 5% CO_2_ incubator. The plates with differentiated THP1 cells were wash with serum-free medium. For THP1 toxicity assay, the diluted test samples (extracts or pure compounds) were add over differentiated THP1 cells and plates were incubated during 48 h at 37 °C in a 5% CO_2_ incubator. Cell growth was determined by the Alamar Blue assay. IC_50_ and IC_90_ values were calculate from dose response curves using XLFit® [32].

## Results and Discussion

### Essential Oil Composition

In this study, the yield (0.34 ± 0.001%) of *S. ceratophylla* EO was higher than in other studies performed in the middle of Jordan, where the yield was 0.1% [15]. It is also lower than those grown in Turkey with a yield of 0.4% [13] and 0.8% [3] and in Iran (0.6%, [12]). Usually, the yield variation of *S. ceratophylla* EO is relate to the influence of environmental factors linked to predominant climatic and geographical differences in various growth areas [34, 35].

The EO was analyzed by GC and GC/MS, the identified components and their relative percentages are listed in Table 1. Seventy-one components were identified, representing 93.5% of the total constituents (Table 1). The major compounds of the EO were linalool (54.8%), germacrene-D (6.4%), bicyclogermacrene (5%) and the bicyclic diterpene alcoholsclareol (4.3%). As far as we know, there is no previous report on the *S. ceratophylla* EO composition originated from Dana Biosphere Reserve (Southern Jordan). However, there are some works reporting the chemical composition of *S. ceratophylla* originated from middle of Jordan [15], Turkey [3, 13, 14] and Iran [12].

**Table 1:** Composition of *Salvia ceratophylla* L*.* essential oil from Dana Biosphere Reserve, South Jordan

No.	RRI	Compounds	%
	969	β-Pinene	0.1
	981	β-Myrcene	0.1
	1010	1,8-Cineole	2.0
	1019	*trans*-Ocimene	0.1
	1029	*cis*-Ocimene	0.4
	1038	γ-Terpinene	0.1
	1045	*trans*-Linalool oxide	0.2
	1058	*cis*-Linalool oxide	0.2
	1074	Linalool	54.8
	1128	δ-Terpineol	0.1
	1142	Terpinen-4-ol	0.1
	1152	α-Terpineol	1.2
	1192	*cis*-Geraniol	0.1
	1217	Geraniol	0.5
	1224	Linalyl acetate	0.2
	1319	δ-Elemene	0.4
	1331	Neryl acetate	0.2
	1333	α-Cubebene	0.1
	1350	Geranyl acetate	0.2
	1361	α-Copaene	1.2
	1369	β-Bourbonene	0.2
	1375	β-Elemene	1.2
	1397	α-Gurjunene	0.2
	1403	Aristolene	1.0
	1404	β-Caryophyllene	1.9
	1415	β-Copaene	0.1
	1425	α-Bergamotene	0.5
	1426	Aromandendrene	0.1
	1438	Isoledene	0.1
	1440	Humulene	0.1
	1448	Alloaromadendrene	0.1
	1462	γ-Gurjunene	0.1
	1465	γ-Muurolene	0.2
	1467	Guaia-1(10),11-diene	0.2
	1470	Germacrene D	6.4
	1475	β-Selinene	0.1
	1480	δ-Selinene	0.4
	1483	Cubebol	0.1
	1487	Bicyclogermacrene	5.0
	1491	α-Muurolene	0.1
	1493	δ-Cadinene	0.1
	1505	γ-Cadinene	0.2
	1511	*cis*-α-Santalol	0.1
	1551	β-Maaliene	0.1
	1560	Maaliol	0.1
	1565	Palustrol	0.2
	1568	Spathulenol	2.4
	1575	Caryophyllene oxide	0.5
	1580	Globulol	0.5
	1585	Salvial-4(14)-en-1-one	0.1
	1589	Viridiflorol	0.3
	1592	Cubeban-11-ol	0.1
	1600	β-Eudesmol	0.1
	1603	Ledol	0.1
	1612	Neointermedeol	0.1
	1622	Junenol	0.1
	1628	Isospathulenol	0.3
	1633	Di-epi-1,10-cubenol	0.1
	1646	*T*-Muurolol	0.6
	1650	δ-Cadinol	0.1
	1652	Cubenol	0.1
	1658	Aromadendrene oxide	0.3
	1661	α-Cadinol	1.0
	1679	Isoaromadendrene epoxide	0.1
	1688	Ledene oxide	0.1
	1707	Shyobunol	0.4
	1727	8-Hydroxylinalool	0.1
	1984	Manoyl oxide	0.7
	2031	Epimanool	0.5
	2133	*trans*-Geranylgeraniol	0.1
	2166	Sclareol	4.3
		Total	93.5

Compounds listed in order to their elution on the HP-1 column

RRI Relative retention indices calculated against C_8_ – C_23_*n*-alkanes on the apolar HP-1 column

% calculated from GC/MS data

The EO of the dried flowers of *S. ceratophylla* grown in the middle of Jordan was dominate by oxygenated monoterpenes (58.28%) (Al Jaber, 2016). *S. ceratophylla* oil grown in Elazığ, Turkey is characterized by a high amount of germacrene D (27.4%) [13].

Gürsoy et al. [3] also reported that the composition of *S. ceratophylla* EO grown in Turkey, was dominated by γ-muurolene (11.4%) and α-pinene (7.6%) as major compounds. Furthermore, the oils of *S. ceratophylla* grown in Kayseri, Elazığ and Adıyaman, Turkey were analyzed: α-Pinene (27.0%), β-pinene (16.3%) and β-caryophyllene (10.6%) were the major components in the Kayseri sample, α-pinene (24.6%) and β-pinene (10.3%) in the Elazığ samples, α-pinene (23.7%), 1,8-cineole (8.9%) and borneol (7.0%) in the Adıyaman sample. The *S. ceratophylla* EO from Iran was dominated by cis-thujopsene (10.9%), cyclopentadecanolide (6.1%), methyl linoleate (4.5%) and α-humulene (3.6%) [12].

Our results revealed that *S. ceratophylla* EO, originated from southern Jordan showed significant differences compared with the oils obtained from samples grown in other locations and was distinguished by its high concentration of linalool. Our results also showed that this EO is distinct by containing the bicyclic diterpene alcohol sclareol, while this compound was absent in *S. ceratophylla* from Middle Jordan [35], Turkey [3, 13, 14] and Iran [12]*.* This variation might be ascribed to the influence of various factors such as seasonal variation and some environmental conditions such as climate, heavy metals pollution and type of soil, in which the plants were grown [35, 36]. Furthermore, harvesting time, temperature and drying period of plants, may also influence the yield of the EO and their phytochemical composition, because these factors are known to increase the concentration of some aromatic oil components, especially linalool [37]. Kumar et al. [38] reported higher contents of linalool in *S. sclarea* under high daily light exposure, while the highest content of germacrene D and sclareol required 50% and 75% of shade, respectively.

Moreover, the EO of other *Salvia* species grown in Jordan were dominate by linalool as major component. Linalool was the major component in the oils of fresh and dry aerial parts of *S. dominica* (31.4% and 18.3%, respectively) [39]. The main compound of EO extracted from fresh *S. verbenaca* was mainly linalool [40].

### Cytotoxic Activity Against Cancer Cells

The cytotoxic activity of *S. ceratophylla* EO and other extracts against cancer and non-cancer cell lines is summarize in Table 2. To the best of our knowledge, the cytotoxic activity of *S. ceratophylla* EO is reporting here for the first time. The EO (up to a concentration of 100 µg/mL), linalool and other tested pure compounds (up to a concentration of 10 µg/mL) did not exhibit cytotoxic activities against all cell lines tested. In agreement with our results, other reports showed that linalool did not exhibit cytotoxic activity up to a concentration of 200 μg/mL against several cancer cell lines [41]. Nevertheless, linalool had significant cytotoxic activity against various cancer cell lines, such as BCC-1/KMC, AGS, RTCC-1/KMC, U2OS, HeLa, H520, H661, OSCC-1/KMC, J82 [42]. Furthermore, it enhanced the cytotoxic activity of doxorubicin against doxorubicin-resistant MCF-7 breast cancer cells [43].

**Table 2:** Cytotoxic activity (IC_50_) of *S. ceratophylla* EO, polar and non-polar extracts and pure compounds

Sample code	Cancer cell lines				Kidney epithelial	Kidney fibroblast
	SK-MEL	KB	BT-549	SK-OV-3	LLC-PK1	Vero
	IC_50_	IC_50_	IC_50_	IC_50_	IC_50_	IC_50_
	µg/mL	µg/mL	µg/mL	µg/mL	µg/mL	µg/mL
EO	NA	NA	NA	NA	NA	NA
A	95	70	70	100	60	> 100
B	> 100	NA	> 100	NA	NA	NA
C	NA	NA	NA	NA	NA	NA
D	NA	NA	NA	NA	NA	NA
Linalool	NA	NA	NA	NA	NA	NA
(-)trans-Caryophyllene	NA	NA	NA	NA	NA	NA
α-Terpineol	NA	NA	NA	NA	NA	NA
Doxorubicin	0.8	1.4	0.85	2	1.2	> 5

*NA* not active at 10 µg/mL for tested pure compound, and at 100 µg/mL for tested extracts

In addition, among all *S. ceratophylla* polar and nonpolar extracts tested, the hexane extract (A) exhibited slight cytotoxic activity against all tested cancer cell lines. The IC_50_ values of A extract against SK-MEL, KB, BT-549, SK-OV-3, LLC-PK1 and VERO cells were 95, 70, 70, 100, 60, and > 100 µg/mL, respectively.

However, our results are in agreement with other previous reports. Abu-Dahab et al. [16] showed that 70% ethanol extract of *S. ceratophylla* originated from Middle Jordan, exhibited low cytotoxic activity against several cancer cell lines. Furthermore, compared with other *Salvia* species from Jordan, the cytotoxic activity of *S. ceratophylla* 70% ethanol extract tested by using the sulforhodamine B colorimetric assay was comparable or even higher against the obesity-related HCT116 and Caco2 colorectal carcinoma cell lines. The cytotoxic activity of *S. ceratophylla* against other obesity-related colorectal cell lines including HT29, SW620, and PDL was low, but higher than other *Salvia* species tested [5, 17] demonstrated that among various species of *Salvia*, *S. ceratophylla* methanol extract exhibited the strongest activity against human amelanotic melanoma (C32, CRL1585), and renal cell adenocarcinoma (ACHN, CRL1611) with IC_50_ values of 20.8 and 27.2 μg/mL, respectively.

However, the variation in the cytotoxic activity of *Salvia* species may be due to the variation in their chemical composition, which can be influence by various factors such as chemotype and environment [4]. It was also reported that the cytotoxic activity of *S.officinalis* was affect by solvents used to obtain various extracts with various yields and quality of secondary metabolites [44].

### Inhibition of Oxidative Stress and Anti-Inflammatory Activity

Nitric oxide (NO) is a free radical and its production at high levels was associated with inflammation and cancer. The metabolic pathway, which is known as the L-arginine: NO pathway is the main source for NO production in mammalian cells by a group of enzymes known as the nitric oxide synthases (NOS). Inducible NOS (iNOS; NOS2; or type II NOS) is the enzyme responsible for the involvement of NO in inflammatory processes. Accordingly, the inhibition of NO production has been considered as a potential anti-inflammatory and cancer chemo-preventive strategy [45]. On the other hand, the activation of NF-κB represents a mechanistic connection between chronic inflammation and tumorigenesis [46]. The Specificity Protein 1 (Sp1) is a transcription factor, which binds GC/GT-rich promoter elements through three C2H2-type zinc fingers that are present in their C-terminal domains [47]. Sp1 plays a major role in regulating the expression of genes involved in cell differentiation, cell cycle and apoptosis [48]. As compared with normal tissues, the activity and / or expression levels of Sp 1 were increased in some tumors such as carcinomas of breast [49], thyroid [50], and pancreas [51]. Sp1 plays an important regulatory role in controlling pathways of tumor development and progression. Thus, it may be an attractive therapeutic target for anticancer natural agents.

The EO of several *Salvia* species were being report to inhibit iNOS [52]. Therefore, we also evaluated, for the first time, the in vitro inhibiting intracellular oxidative stress activity of the EO and polar and nonpolar extracts *S. ceratophylla* from southern Jordan*.*

With exception of the EO, which did not decrease the oxidative stress, our results revealed that all other extracts exhibited antioxidant activity by inhibiting intracellular oxidative stress (Table 3). The hexane (A) and ethyl acetate (B) extracts were the most active in decreasing oxidative stress. At the concentration 1000 µg/mL the decrease by the extracts was 50 and 47%, respectively, while at the concentration 500 µg/mL the decrease by the extracts was 56 and 25%, respectively. Among all tested pure compounds, only caryophyllene showed weak activity in decreasing oxidative stress by 35 and 31% at concentrations of 500 and 250 µg/mL, respectively.

**Table 3:** Anti-inflammatory activity of *S. ceratophylla* essential oil, other extracts and pure compounds

Sample code	% decrease in	Oxidative stress	NO inhibition	NF- κB inhibition	Sp-1 inhibition
	1000 µg/mL	500 µg/mL	IC_50_ (µg/mL)	IC_50_ (µg/mL)	IC_50_ (µg/mL)
EO	NA	NA	90	38	NA
A	50	47	32	70	70
B	56	25	30	85	160
C	43	29	NA	NA	NA
D	44	21	40	150	NA

	500 µg/mL	250 µg/mL			
Linalool	NA	NA	18	60	NA
Caryophyllene	35	31	NA	45	48
α-Terpineol	NA	NA	NA	100	NA
Quercetin 25 µM	75	–	–	–	–
Parthenolide	–	–	0.48	0.33	–

*NA* Not active at tested concentration

The EO showed the lowest inhibition in NO production with an IC_50_ 90 µg/mL and the highest inhibition activity against NF-κB with an IC_50_ 38 µg/mL. At the same time, it did not inhibit the transcription factor Sp-1. The extracts A, B and D inhibited NO production and NF- κB with IC_50_ values of 32, 30, and 40 µg/mL, and 70, 85 and 150 µg/mL, respectively. The two non-polar extracts (hexane and ethyl acetate, A and B) of *S. ceratophylla*, inhibited Sp-1 with IC_50_ values of 70 and 160 µg/mL, respectively.

Linalool was the only pure compound showing high and significant NO inhibition with an IC_50_ value of 18 µg/mL. Linalool, caryophyllene and α-terpineol inhibited NF-κB activity with IC_50_ values of 60, 45 and 100 µg/mL, respectively. Caryophyllene was the only pure compound, which inhibited Sp-1 with an IC_50_ value of 48 µg/mL.

The antioxidant activity of *S. ceratophylla* EO measured by different methods was moderate and slightly lower than the activity of *S. palaestina* essential oil [3]. Moreover, *S. officinalis* EO from Jordan showed anti-inflammatory effects and significantly inhibited NO production stimulated by LPS in macrophages, without affecting cell viability, in concentrations up to 0.64 μL/mL [53].

Nevertheless, the anti-inflammatory activity assessed by edema reduction was observed for *S. ceratophylla* ethylacetate extract with an ID_50_ 98 μg/cm^2^ compared with indomethacin, the hexane and methanol extract showed very low activity with an ID_50_ of > 300 μg/cm^2^ [6]. Moreover, using the FRAP method, *S. ceratophylla* exhibited significant antioxidant activity 290.7 μmol FeSO_4_ eq/g of dried extract [17].

However, the previously obtained results showed that linalool significantly inhibited carrageenin-induced hind paw edema (55%, *p* = 0.03) at a dose of 25 mg/kg [54]. Ma et al. [55] reported that the effect of linalool on cigarette smoke induced acute lung inflammation and showed that it suppressed the activation of NF-κB in a dose-dependent manner. Hassan et al. [46] showed that α-terpineol inhibited the growth of cell lung carcinoma through significant inhibition of the NF-κB pathway.

However, in agreement with Gürsoy et al. [3] it is important to mention that the anti-oxidative activity of EOs or extracts were link with the polarity of their chemical constituents. EOs with non-polar secondary metabolites such as terpenoids, were minimally or not active. The anti-inflammatory and radical scavenging activity of *S. ceratophylla may* be ascribe to the high total phenolic contents, thus contributing to their electron transfer/hydrogen donating capability [3, 17, 56]*.*

### Antileishmanial and Antitrypanosomal Activities

The present study provided for the first time, data on the activity of *S. ceratophylla* EO and other extracts (from Jordan) against *T. brucei* and *L. donovani* in promastigote and amastigote stages. Furthermore, this study demonstrated the antiprotozoal activity of pure compounds (linalool, α–terpineol and β-caryophyllene).

EO and extracts of *S. ceratophylla* and pure compounds did not exhibit antileishmanial activity against extracellular (promastigote) and intracellular (amastigote) forms of *L. donovani* at all tested concentrations (Table 4). On the other hand, the Jordan *S. ceratophylla* exhibited significant antitrypanosomal activity against *T. brucei.* The EO showed higher antitrypanosomal activity with IC_50_ and IC_90_ values of 2.65 and 3.61 µg/mL, respectively. The inhibiting activity of the EO was better than that of the control (α-difluoromethylornithine) with IC_50_ and IC_90_ values of 4.792 and 7.76 µg/mL. Among all extracts, the Ethyl acetate extract (B) was the only one that exhibited moderate antitrypanosomal activity against *T. brucei* with IC_50_ and IC_90_ values of 15.78 and 19.28 µg/mL, respectively. Among the pure compounds, α-terpineol was the only compound that exhibited antitrypanosomal activity against *T. brucei* similar to the control (α-difluoromethylornithine) with IC_50_ and IC_90_ values of 6.04 and 8.22 µg/mL, respectively.

**Table 4:** Antiprotozoal activity of *S. ceratophylla* essential oil, other extracts and pure compounds (only the antitrypanosomal activity)

Tested sample	*L. donovani Promastigote*		*L. donovani Amastigote*		*L. donovani Amastigote* + THP1		*T. brucei*		THP1 cells toxicity		Tested concentr-ations (µg/mL)
	IC_50_	IC_90_	IC_50_	IC_50_	IC_50_	IC_90_	IC_50_	IC_90_	IC_50_	IC_90_	
EO A B C D Linalool α -terpineol β-Caryophyllene Amphotericin B Pentamidine α-difluorome-thylornithine	NA NA NA NA NA NA NA NA 0.140 1.024 NT	NA NA NA NA NA NA NA NA 0.324 9.280 NT	NA NA NA NA NA NA NA NA 0.181 3.797 NT	NA NA NA NA NA NA NA NA NA NA T	NA NA NA NA NA NA NA NA NA NA NT	NA NA NA NA NA NA NA NA NA NA NT	2.65 NA 15.78 NA NA NA 6.04 NA NT NT 4.792	3.65 NA 19.28 NA NA NA 8.22 NA 0.426 – 7.760	NA NA NA NA NA NA NA NA 0.375 – NT	NA NA NA NA NA NA NA NA 0.190 1.735 NT	20–0.8 20–0.8 20–0.8 20–0.8 20–0.8 10–0.4 10–0.4 10–0.4 2.0–0.08 10–0.4 20–0.8

*NA* not active at tested concentration, *NT* not tested against listed protozoans

Interestingly, the results of human acute monocytic leukemia cells toxicity (THP1) showed that the *S. ceratophylla* EO, Ethyl acetate extract (B) and α-terpineol did not exhibit cytotoxic effects on mammalian cells at the concentrations tested.

Other authors stated that extracts obtained from other *Salvia* species led to similar results than those of *S. ceratophylla* extracts, such as the activity of *S. officinalis* methanol extract from leaves against promastigote and amastigote forms of *L. major* [57]. Moreover, the liquid form of *S. officinalis* extract distributed in the German market did not show remarkable antileishmanial activity against *L. donovani* [21].

Morales et al. [25] studied the antileishmanial activity of various EO components such as linalool, carvacrol, geraniol and terpinen-4-ol against *L. infantum* promastigotes. Accordingly, among the compounds tested, carvacrol exhibited the highest activity at the lowest concentration of 6.25 µg/mL followed by linalool, which exhibited dose-dependent antileishmanial activity. At a concentration of 37.5 µg/mL the inhibitory activity of linalool was similar to the control drug (Pentamidine). Moreover, linalool, the main EO component of *Croton cajucara* reduced the viability of *L. amazonensis* promastigotes at very low concentrations (MIC, 85.0 pg/ml) and presented no cytotoxic effects against mammalian cells [24].

Ślusarczyk et al. [63] showed that among 880 plant and fungal extracts, the dichloromethane extract of *S. miltiorrhiza* was the only extract that inhibited 97% of *T. brucei rhodesiense* growth at the lowest concentration tested (0.81 μg/mL). Accordingly, the antitrypanosomal activity of *S. miltiorrhiza* dichloromethane extract was correlate with the presence of tanshinone-type diterpenoids, which also exhibited antitrypanosomal activity with IC_50_ values of 0.5 µM to over 30 µM against *T. brucei rhodesiense* [58].

Both, minor compound (nerolidol) and major compound (linalool) of *Strychnos spinosa* EO demonstrated high and selective activity towards *Trypanosoma* (SI = 35.7 and > 40, respectively, and IC_50_ = 1.7 and 2.5 µg/mL, respectively). α-Terpineol was slightly active against *T. brucei brucei* blood stream forms [59]. However, these authors also reported that the discrepancy in the results might be due to different used methods and/or due to differences in stereoisomeric forms of the compounds tested.

## Conclusion

The present study revealed that *S. ceratophylla* EO from Jordan was characterize by a high amount of linalool. Additionally, the EO exhibited significant antitrypanosomal activity against *T. brucei.* Moreover, the inhibitory activity of *S. ceratophylla* EO against *T. brucei* was stronger than that of the control (α-difluoromethylornithine).

This study highlights the potential of this EO as a good candidate for the treatment of the hard to cure neglected trypanosomiasis infections, also it is demonstrating that *S. ceratophylla* bears promising therapeutic potential for further drug development. Importantly, this work also clearly validated some of the traditional medicinal uses of *Salvia* species.
